# Plant-based dietary patterns and ultra-processed food consumption: a cross-sectional analysis of the UK Biobank

**DOI:** 10.1016/j.eclinm.2024.102931

**Published:** 2024-11-14

**Authors:** Kiara Chang, Jennie C. Parnham, Fernanda Rauber, Renata B. Levy, Inge Huybrechts, Marc J. Gunter, Christopher Millett, Eszter P. Vamos

**Affiliations:** a Public Health Policy Evaluation Unit, School of Public Health, Imperial College London, London, United Kingdom; b Department of Preventive Medicine, School of Medicine, University of São Paulo, São Paulo, Brazil; c Center for Epidemiological Research in Nutrition and Health, University of São Paulo, São Paulo, Brazil; d Nutrition and Metabolism Branch, International Agency for Research on Cancer, Lyon, France; e Department of Epidemiology and Biostatistics, School of Public Health, Imperial College London, London, United Kingdom; f NOVA National School of Public Health, Public Health Research Centre, Comprehensive Health Research Center, CHRC, NOVA University Lisbon, Portugal

**Keywords:** Ultra-processed foods, Plant-based diet, Flexitarian, Vegetarian, Vegan

## Abstract

**Background:**

Dietary shift towards more plant-based options is increasingly popular, but the quantity of ultra-processed foods (UPFs) they contain is largely unknown. This study assessed the level of UPF and minimally processed food consumption among regular and low red meat eaters, flexitarians, pescatarians, vegetarians and vegans in a large dataset of United Kingdom (UK) adults.

**Methods:**

This is a cross-sectional analysis of the UK Biobank participants recruited between December 19, 2006, and October 1, 2010. Responses to food frequency questions were used to identify diet types for vegans (never eating any animal-based foods); vegetarians (never eating meat/fish); pescatarians (never eating meat); flexitarians (consumed fish/meat under twice a week); low red meat eaters (consumed fish/poultry more than once a week but red/processed meat under twice a week); and regular red meat eaters (consumed red/processed meat more than once a week). Consumption of all food and drinks collected in 24-h recalls between April 29, 2009, and June 28, 2012, were categorised using the Nova classification. The primary outcomes are the consumption of UPFs and minimally processed foods, expressed as a percentage of daily food intake (grams/day). Multivariable linear regression assessed the mean percentage point difference in UPF and minimally processed food consumption between diet types.

**Findings:**

This study included 199,502 UK Biobank participants (mean age 58.2 [standard deviation 7.9] years; 55.1% women). The mean UPF consumption was 24.2%, 21.9%, 22.0%, 20.4%, 23.8%, and 22.7% among 75,091 regular red meat eaters, 70,144 low red meat eaters, 45,057 flexitarians, 4932 pescatarians, 4119 vegetarians and 159 vegans, respectively. The adjusted results suggested that compared with regular red meat eaters, UPF consumption was 1.3 percentage points higher among vegetarians (95% confidence interval [CI]: 0.9, 1.7) and lower among low red meat eaters (−1.3, 95% CI: −1.4, −1.1), flexitarians (−0.8, 95% CI: −1.0, −0.7), and pescatarians (−1.6, 95% CI: −1.9, −1.2). The UPF consumption in vegans were not significantly different from regular red meat eaters (1.2 percentage points, 95% CI: −0.7, 3.2). Minimally processed food consumption was higher in all other types of diet than regular red meat eaters, with an adjusted percentage point difference ranged from 0.4 (95% CI: 0.005, 0.9) for vegetarians to 3.2 (95% CI: 1.0, 5.5) for vegans compared with regular red meat eaters.

**Interpretation:**

This UK-based study found higher UPF consumption in vegetarian diets and lower in diets with a modest amount of meat or fish. It is important that policies which encourage the urgently needed transition to more sustainable dietary patterns also promote rebalancing diets towards minimally processed foods.

**Funding:**

10.13039/501100000272National Institute for Health and Care Research (NIHR) 10.13039/501100012349School for Public Health Research, 10.13039/501100000321World Cancer Research Fund.

**Research in context**

**Evidence before this study**

We searched PubMed from the inception of the database to May 6, 2024, for studies published that have assessed ultra-processed food (UPF) consumption in two or more diet types with different levels of animal-sourced food avoidance (e.g., vegetarians, meat eaters). No language restrictions were applied. Only two studies were identified and these were smaller in sample size compared with our study. No previous study has been conducted on a United Kingdom (UK)-based sample despite the UK being one of the leading consumers of UPFs globally. Given the environmental impacts of animal-sourced foods, it is important to understand how the consumption of UPFs and minimally processed foods varies across dietary patterns of animal-sourced food consumption.

**Added value of this study**

This is the first UK study and the largest study conducted to the best of our knowledge. We found using data from the UK Biobank study that the consumption of UPFs was high and represented more than 20% of daily food intake and more than 46% of daily energy intake in all types of diet. Compared with diets of regular red meat eaters, vegetarians consumed a significantly higher amount of UPFs while diets of low red meat eaters, flexitarians, and pescatarians showed the opposite.

**Implications of all the available evidence**

The consumption of UPFs is universally high across all types of dietary patterns based on animal-sourced food avoidance. The adaptation of plant-based meat and dairy alternatives is evident particularly in the diets of pescatarians, vegetarians, and vegans.

## Introduction

The global temperature has risen to 1.3 °C above pre-industrial levels and fast approaching the 1.5 °C target set in the Paris Agreement.^1^ Dietary shift towards more plant based options is increasingly common partly due to considerations for planetary health, as meat and dairy are widely understood as the biggest contributors to greenhouse gas emissions from individual diets.^2^^,^^3^ In the United Kingdom (UK), representative surveys showed that 4% and 1% of the respondents identified as vegetarian and vegan respectively, and another 10% indicated being mainly vegetarian but occasional meat consumer.^4^ Market research data for Western Europe suggested that between 2007 and 2022, the per capita sales volume of milk and milk drinks reduced by 3.9 kg while plant-based milk increased by 1.2 kg.^5^ Notably, the sales volume of meat substitutes grew by 10.1% from 2021 to 2022 while processed meat sales declined by 2.5%.^5^ These data are supportive of a concurrent shift towards adaptation of a more plant-based diet, and raise concerns about their quality and healthfulness in light of the increased traction of plant-sourced but highly industrially processed products.^6^

The United Nations has recommended a sustainable healthy diet consisting of a diverse range of minimally processed foods while avoiding ultra-processed foods (UPFs).^7^ Based on the Nova classification, UPFs are industrial products made through a sequence of extensive industrial processes that include fractioning of original foods into substances and often chemically modifying them, then recombining the substances into products during which various additives are frequently used.^8^ UPFs typically contain little or no whole foods, and are higher in calorie, salt, fat, and sugar content but lower in fibre.^8^ Most industrially produced meat and dairy substitutes are UPFs, as are soft drinks, mass-produced bakery goods and most breakfast cereals. They are made hyper-palatable, relatively cheap and convenient, heavily marketed and purposefully designed to displace minimally processed foods in diets.^8^

The consumption of UPFs is rising globally and has reached beyond 50% of daily calorie intake in the UK and United States (US).^9^^,^^10^ The causal link between UPF consumption and weight gain was demonstrated in a randomised controlled trial, and large-scale cohort studies have generally suggested an association between higher UPF consumption and increased risk of cardiometabolic diseases, cancer, and mortality.^11^, ^12^, ^13^, ^14^, ^15^ A recent prospective cohort study from the UK has shown that diets high in plant-sourced UPFs were linked to increased cardiovascular risk.^16^ However, data are scarce on the level of UPFs and minimally processed foods consumed by people adhering to various dietary patterns according to differing levels of animal-sourced food avoidance. This is an important gap in knowledge as the UPF industry is well-aware of the increasing demand of plant-sourced foods, and the production and marketing of plant-based alternatives (including meat, seafood, eggs, and dairy) have greatly increased in the past decade.^17^ Therefore, this study aims to quantify and compare the degrees of industrial food processing in dietary patterns of regular red meat eaters, low red meat eaters, flexitarians, pescatarians, vegetarians and vegans in a large sample of middle-aged adults who participated in the UK Biobank.

## Methods

### Data source and study population

The UK Biobank is a large prospective cohort study that recruited half a million participants aged between 40 and 69 years old identified from the National Health Service patient registry.^18^ Study participants attended one of the 22 assessment centres across England, Wales, and Scotland for the collection of baseline data between December 19, 2006 and October 1, 2010. They completed questionnaires covering socio-demographic, lifestyle (including smoking status and a 29-item short food frequency questionnaire [FFQ]) and psychosocial characteristics at recruitment.^18^ Physical measurements were obtained using standardised procedures, and medical history and medication use were verified by trained research staff.

**Fig. 1:**
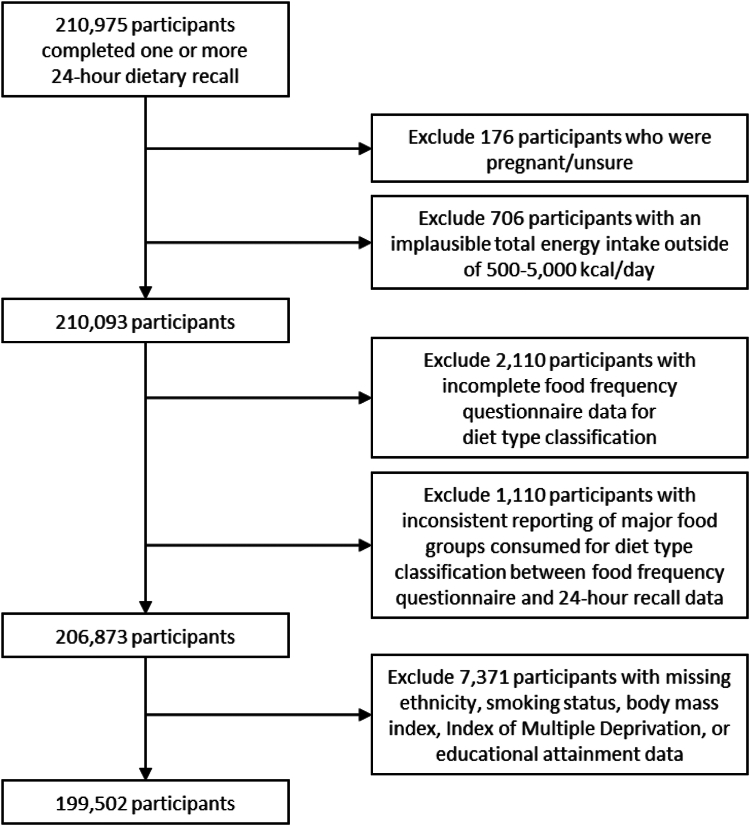
**Flowchart for the derivation of study population from the UK Biobank**.

In a subsequent addendum of the UK Biobank protocol, a web-based 24-h dietary recall (Oxford WebQ) was introduced in 2009 and completed by the last 70,000 study participants attending baseline assessment. The same 24-h recall was sent out to all participants with a valid email address (about 320,000 participants) on four separate occasions during 2011–2012. The dietary recall was designed to capture the consumption of over 200 food and beverage items in the previous 24 h, and has been validated showing similar energy and nutrient intakes as an interviewer-administered 24-h recall.^19^ We considered a total of 210,975 participants with one or more 24-h recall data collected between April 29, 2009 and June 28, 2012 for inclusion in this study (Fig. 1).

### Outcome measures

Participants' 24-h dietary recall data were used to construct the dietary outcome measures. We applied the Nova classification assigning each food and beverage item collected by the 24-h recall to one of the four Nova food groups based on the extent and purpose of industrial food processing they underwent.^8^ Nova 1 includes unprocessed and minimally processed foods that are edible parts of food as found in nature or have undergone minimal processing without adding any substances (e.g., salt, sugar) to the original food. Examples of Nova 1 foods are fruits, vegetables, legumes, roots and tubers, milk and meat. Nova 2 includes processed culinary ingredients such as sugar, vegetable oils and butter. Nova 3 represents processed foods that are manufactured by combining Nova 1 food with Nova 2 ingredients, e.g., canned vegetables in brine, freshly made breads and cheeses. Nova 4 represents UPFs that are industrial formulations made often with many substances that are exclusively available for industrial use (e.g., modified starches, protein isolates, high-fructose corn syrup, emulsifiers, thickening agents) and undergo extensive food processing procedures that cannot be conducted in a domestic kitchen. Examples of Nova 4 foods are soft drinks, mass-produced industrial-processed breads, sweet or savoury packaged snacks, breakfast ‘cereals’, reconstituted meat products and ready-to-eat/heat food. The methodological details have been previously described.^12^^,^^13^

We averaged dietary intakes across multiple 24-h recalls. We assessed for primary outcomes the participants' consumption of foods from each Nova group measured as a proportion of total daily food/energy consumed (%g/day and %kcal/day, respectively). The secondary outcomes were the participants' absolute daily intake of food/energy from each Nova group (g/day and kcal/day, respectively). We combined the dietary intakes of Nova 1 and 2 foods in one measurement as they are mostly consumed in combination, and refer to ‘minimally processed foods’ from here onwards as they represent the largest quantity in diet.

### Diet types of the UK Biobank participants

Study participants were categorised as regular or low red meat eater, flexitarian, pescatarian, vegetarian, or vegan based on their responses to selected FFQ questions relevant for the classification of these diet types. Responses to seven questions were used that queried participants' frequency of consuming oily fish, non-oily fish, processed meat, poultry, beef, lamb, and pork over the past year.^20^ Participants were given the following options to select from: never, less than once a week, once a week, 2–4 times a week, 5–6 times a week, once or more daily, do not know, or prefer not to answer. A further question assessed participants' avoidance of dairy products and eggs or foods containing eggs. Participants who did not respond to all eight FFQ questions and those responded ‘do not know’ or ‘prefer not to answer’ to one of these questions were considered ambiguous for the purpose of diet type classification and were excluded from the study.

Participants were classified as vegans if they reported never eating any type of meat, fish, eggs or dairy products. Vegetarians included lacto- and ovo-vegetarians who reported never eating any type of meat or fish. Pescatarians were identified as those reported eating oily/non-oily fish but never consumed red meat, processed meat, or poultry. Flexitarians were defined as participants reported eating fish, red meat, processed meat, or poultry no more than once a week. The remaining participants were classified as meat eaters and were further divided into two categories. Low red meat eaters were defined as those reported eating red/processed meat no more than once a week (but consumed fish or poultry more than once a week). Regular red meat eaters were frequent consumers reported eating red/processed meat more than once a week.

We cross-compared participants’ diet type with their dietary intake of similar food groups (meat, fish, eggs, and dairy) as recorded in the 24-h recalls, and those with inconsistencies in data reporting between the FFQ and 24-h recalls were excluded from the study. We additionally presented the mean daily intake of the following key nutrients as part of the characteristics for the overall sample and the six diet types: total energy intake (kcal/day), free sugar intake (as a percentage of total energy intake), saturated fatty acids intake (as a percentage of total energy intake), sodium density (mg per 1000 kcal intake), and fibre density (g per 1000 kcal intake).

### Covariates

Study covariates included age, sex (male, female), ethnicity (white, non-white), smoking status (never smoked, ex-smoker, current smoker), physical activity level using the International Physical Activity Questionnaire (low, moderate, high, missing),^21^ body mass index (BMI) categorised as underweight (<18.5 kg/m^2^), normal (18.5–24.9 kg/m^2^), overweight (25.0–29.9 kg/m^2^), or obese (≥30.0 kg/m^2^), highest educational attainment (university degree, other), average annual household income (<£18,000, £18,000-£30,999, £31,000-£51,999, >£52,000, missing), Index of Multiple Deprivation (IMD) quintile, and total energy intake (kcal/day, included when the outcome being assessed was based on food weight). IMD is a composite rank score for each small area in the UK derived based on seven domains of deprivation,^22^ and was mapped to participants' postcode in the UK Biobank. We categorised participants' IMD scores into quintiles. Additional covariates considered in the sensitivity analysis included self-reported diagnosis of diabetes (or regular insulin use), high blood pressure (or regularly taking blood pressure medication), depression, or history of cardiovascular disease (angina, heart attack, and stroke) as verified in a nurse interview. Missing data were under 3.0% of the study population for ethnicity, smoking status, BMI, IMD and education. These were assumed missing at random and were subsequently excluded taking a complete case analysis approach. However, participants with missing physical activity or average household income were considerable (14.8% and 9.1% of the study population, respectively) and there may be common factors related to their missingness, such as participants’ willingness to provide data. Therefore, a missing data category was employed to capture this and preserve sample size.

### Ethics

The UK Biobank received ethical approval from the North West Multi-centre Research Ethics Committee (21/NW/0157), and all study participants provided written informed consent at recruitment.^23^

### Statistics

We compared characteristics of the study population and dietary intake of key nutrients by diet type using analysis of variance for continuous and chi-squared tests for categorical variables. We examined the distribution for each continuous variable graphically as well as assessing their skewness and kurtosis before an analysis of variance test was performed. We also tested using the Kruskal Wallis test which do not impose a normality assumption on the distribution and the results were the same. The mean proportion of daily food/energy intake contributing to the Nova food groups (primary outcomes) and their subsidiary food groups were computed separately and presented graphically for regular red meat eaters, low red meat eaters, flexitarians, pescatarians, vegetarians, and vegans identified. The non-parametric Wilcoxon rank sum tests were used to compare the distribution of primary outcomes and its subsidiary food groups between regular red meat eaters as the reference group and participants of other diet type. The non-parametric tests were adopted because the normality assumption was less likely to be met for the subsidiary food groups with smaller consumption relative to the overall diet.

Multivariable linear regression models were performed to assess whether the mean proportion of daily food/energy intake contributed by each Nova food group differed between diet types while adjusted for age, sex, ethnicity, smoking status, physical activity, BMI category, highest education attainment, average household income, IMD quintile, and total daily energy intake (for the food weight models). Similar regression models were performed for the mean absolute daily food/energy intake contributed by each Nova food groups (secondary outcomes).

Sensitivity analyses were performed on primary outcomes: (i) excluding the BMI variable from the models because an examination of the variance inflation factor (VIF) suggested that BMI may be an influential factor susceptible to multicollinearity (with a VIF>30.0 while all other variables had VIF<2.6); (ii) additionally adjusted for the presence of self-reported diagnosis of diabetes (or regular insulin use), high blood pressure (or regularly taking blood pressure medication), depression, or history of cardiovascular disease; (iii) excluding participants with fewer than two 24-h recalls.

Analyses were conducted using Stata version 15. Statistical tests were two-sided with a p-value < 0.05 considered significant.

### Role of funding source

The funders had no role in study design, data collection, data analysis, interpretation, or writing of the report.

## Results

This study included 199,502 UK Biobank participants after excluding 176 individuals who reported being pregnant or were unsure, 706 individuals with an implausible total energy intake beyond 500–5000 kcal/day,^12^^,^^13^ 2110 individuals with incomplete FFQ data, 1110 individuals with an inconsistent reporting of major food groups consumed between the FFQ and 24-h recalls data, and 7371 individuals with missing covariates data (Fig. 1). The mean age was 58.2 (SD = 7.9) years, 55.1% were women, and 96.0% were of white ethnicity (Table 1). Most participants (72.8%) were frequent meat eaters, one in five were flexitarians (22.6%), and much fewer were pescatarian (2.5%), vegetarian (2.1%), or vegan (0.08%). Pescatarians, vegetarians, and vegans were more likely to be women and younger, had a non-white ethnic background, high physical activity level, BMI in the normal range, completed university education, and reside in a more deprived neighbourhood. On average, regular red meat eaters had the highest total energy and lowest fibre density while the opposite was found for vegans. Moreover, saturated fatty acids intake was highest among regular red meat eaters and flexitarians, and lowest among vegans. Vegetarians had the highest sodium density while vegans had the lowest. The mean free sugar intake was similarly high among regular red meat eaters and vegetarians (13.9% of total energy intake), and was lowest among low red meat eaters (13.1% of total energy intake).

**Table 1:** Characteristics of the study population from the UK Biobank by diet types.

	Overall	Regular red meat eater	Low red meat eater	Flexitarian	Pescatarian	Vegetarian	Vegan	p-value^a^
N (%)	199,502 (100.0)	75,091 (37.6%)	70,144 (35.2%)	45,057 (22.6%)	4932 (2.5%)	4119 (2.1%)	159 (0.08%)	
Age (year)								
Mean (SD)	58.2 (7.9)	58.1 (8.1)	58.4 (7.9)	58.8 (7.7)	56.1 (7.8)	54.8 (7.7)	53.8 (8.0)	<0.001
Sex (n, %)								
Male	89,532 (44.8%)	43,765 (58.2%)	25,329 (33.7%)	17,645 (36.1%)	1362 (27.6%)	1367 (33.1%)	64 (40.2%)	<0.001
Female	109,970 (55.1%)	31,326 (41.7%)	44,815 (59.6%)	27,412 (63.8%)	3570 (72.3%)	2752 (66.8%)	95 (59.7%)	
Ethnicity (n, %)								
White	191,614 (96.0%)	72,514 (96.5%)	67,217 (89.5%)	43,421 (95.8%)	4697 (95.2%)	3617 (87.8%)	148 (93.0%)	<0.001
Non-white	7888 (3.9%)	2577 (3.4%)	2927 (3.8%)	1636 (4.1%)	235 (4.7%)	502 (12.1%)	11 (6.9%)	
Smoking status (n, %)								
Never smoked	112,991 (56.6%)	41,332 (55.0%)	40,494 (53.9%)	25,698 (57.7%)	2828 (57.3%)	2557 (62.0%)	82 (51.5%)	<0.001
Ex-smoker	71,037 (35.6%)	26,873 (35.7%)	25,052 (33.3%)	15,913 (35.7%)	1814 (36.7%)	1323 (32.1%)	62 (38.9%)	
Current smoker	15,474 (7.7%)	6886 (9.1%)	4598 (6.1%)	3446 (6.5%)	290 (5.8%)	239 (5.8%)	15 (9.4%)	
Physical activity (n, %)								
High	66,749 (33.4%)	23,872 (31.7%)	25,085 (33.4%)	14,156 (35.7%)	2010 (40.7%)	1559 (37.8%)	67 (42.1%)	<0.001
Moderate	71,966 (36.0%)	27,351 (36.4%)	24,676 (32.8%)	16,692 (35.1%)	1724 (34.9%)	1462 (35.4%)	61 (38.3%)	
Low	31,210 (15.6%)	12,754 (16.9%)	10,034 (13.3%)	7292 (14.3%)	545 (11.0%)	570 (13.8%)	15 (9.4%)	
Missing	29,577 (14.8%)	11,114 (14.8%)	10,349 (13.7%)	6917 (14.7%)	653 (13.2%)	528 (12.8%)	16 (10.0%)	
Baseline BMI status (n, %)								
Underweight (<18.5 kg/m^2^)	1064 (0.5%)	282 (0.3%)	365 (0.4%)	268 (0.5%)	76 (1.5%)	70 (1.6%)	3 (1.8%)	<0.001
Normal (18.5–24.9 kg/m^2^)	73,437 (36.8%)	23,201 (30.8%)	26,218 (34.9%)	18,981 (37.3%)	2766 (56.0%)	2175 (52.8%)	96 (60.3%)	
Overweight (25–29.9 kg/m^2^)	82,966 (41.5%)	32,779 (43.6%)	29,120 (38.7%)	18,081 (41.5%)	1572 (31.8%)	1364 (33.1%)	50 (31.4%)	
Obese (≥30 kg/m^2^)	42,035 (21.0%)	18,829 (25.0%)	14,441 (19.2%)	7727 (20.5%)	518 (10.5%)	510 (12.3%)	10 (6.2%)	
Highest educational attainment (n, %)								
College/University degree	86,824 (43.5%)	31,090 (41.4%)	29,348 (39.0%)	20,732 (41.8%)	3075 (62.3%)	2483 (60.2%)	96 (60.3%)	<0.001
Other	112,678 (56.4%)	44,001 (58.5%)	40,796 (54.3%)	24,325 (58.1%)	1857 (37.6%)	1636 (39.7%)	63 (39.6%)	
Average household income (n, %)								
>£52,000	57,361 (28.7%)	21,701 (28.8%)	20,514 (27.3%)	12,359 (29.2%)	1551 (31.4%)	1206 (29.2%)	30 (18.8%)	<0.001
£31,000–£51,999	51,920 (26.0%)	19,957 (26.5%)	17,913 (23.8%)	11,568 (25.5%)	1324 (26.8%)	1106 (26.8%)	52 (32.7%)	
£18,000–£30,999	44,219 (22.1%)	16,564 (22.0%)	15,422 (20.5%)	10,321 (21.9%)	1006 (20.3%)	870 (21.1%)	36 (22.6%)	
<£18,000	27,789 (13.9%)	10,396 (13.8%)	9593 (12.7%)	6511 (13.6%)	685 (13.8%)	575 (13.9%)	29 (18.2%)	
Missing	18,216 (9.1%)	6473 (8.6%)	6702 (8.9%)	4298 (9.5%)	366 (7.4%)	362 (8.7%)	12 (7.5%)	
Index of multiple deprivation (n, %)								
Quintile 1 (Least deprived)	44,711 (22.4%)	16,808 (22.3%)	15,782 (21.0%)	10,400 (22.4%)	988 (20.0%)	713 (17.3%)	20 (12.5%)	<0.001
Quintile 2	44,436 (22.2%)	16,460 (21.9%)	15,988 (21.2%)	10,177 (22.7%)	1011 (20.4%)	778 (18.8%)	22 (13.8%)	
Quintile 3	41,830 (20.9%)	15,685 (20.8%)	14,857 (19.7%)	9319 (21.1%)	1044 (21.1%)	890 (21.6%)	35 (22.0%)	
Quintile 4	38,467 (19.2%)	14,331 (19.0%)	13,402 (17.8%)	8763 (19.1%)	997 (20.2%)	942 (22.8%)	32 (20.1%)	
Quintile 5 (Most deprived)	30,058 (15.0%)	11,807 (15.7%)	10,115 (13.4%)	6398 (14.4%)	892 (18.0%)	796 (19.3%)	50 (31.4%)	
Total energy intake (kcal/day)								
Mean (SD)	2044.8 (611.7)	2166.3 (632.3)	1971.7 (584.0)	1973.9 (585.6)	1963.3 (597.5)	1957.7 (633.3)	1845.0 (621.6)	<0.001
Free sugar intake (% of total energy intake)								
Mean (SD)	13.6 (7.3)	13.9 (7.2)	13.1 (7.2)	13.7 (7.3)	13.2 (7.5)	13.9 (7.5)	13.3 (8.5)	<0.001
Saturated fatty acids intake (% of total energy intake)								
Mean (SD)	10.8 (3.4)	11.1 (3.4)	10.3 (3.3)	11.1 (3.4)	10.6 (3.5)	10.8 (3.7)	6.7 (2.4)	<0.001
Sodium density (mg/1000 kcal)								
Mean (SD)	932.8 (258.6)	957.3 (258.7)	910.2 (257.4)	922.2 (254.7)	939.5 (266.1)	979.1 (262.4)	882.0 (296.1)	<0.001
Fibre density (g/1000 kcal)								
Mean (SD)	12.6 (5.0)	11.8 (4.8)	13.0 (5.0)	12.6 (5.0)	15.0 (5.2)	16.0 (5.7)	20.9 (6.6)	<0.001

SD, standard deviation; N, sample size.

a p-values were from the comparison of characteristics between diet types were assessed by analysis of variance for continuous variables and chi-squared tests for other categorical variables.

### Dietary intakes based on the percentage of total food (g/day) consumed

**Fig. 2:**
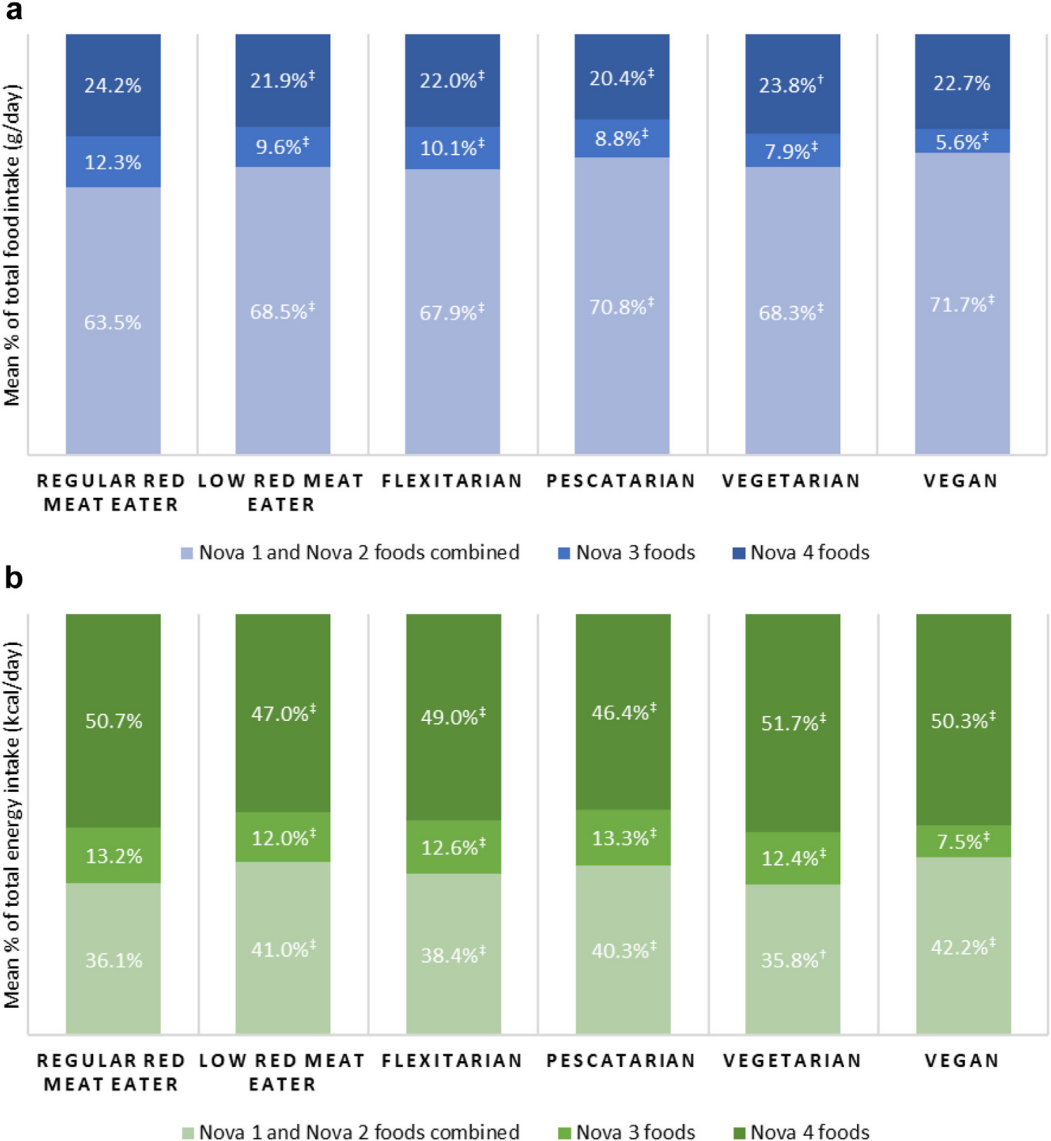
**Mean proportion of the total diet from each Nova food group as measured by: a) daily food intake; and b) daily energy intake.** Nova 1 includes unprocessed and minimally processed foods, Nova 2 includes processed culinary ingredients, Nova 3 represents processed foods, Nova 4 represents ultra-processed foods. ^†^p < 0.05; ^‡^p < 0.01 from ranksum test comparing distribution of consumption against regular red meat eaters.

**Fig. 3:**
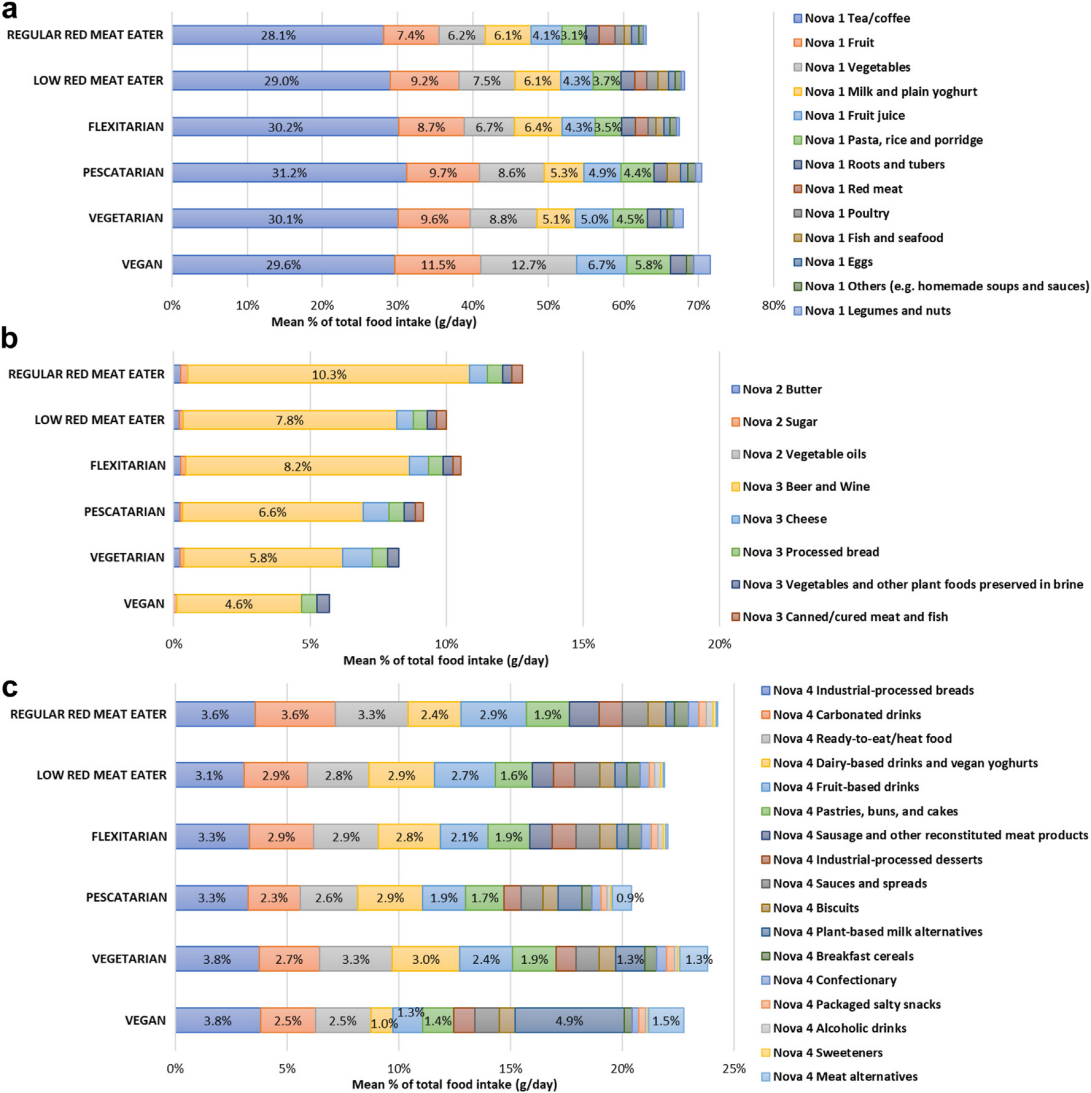
**Mean proportion of daily food intake from subsidiary food groups of: a) Nova 1; b) Nova 2 and Nova 3; and c) Nova 4 food group.** Nova 1 includes unprocessed and minimally processed foods, Nova 2 includes processed culinary ingredients, Nova 3 represents processed foods, Nova 4 represents ultra-processed foods.

Overall, there were no clear patterns observed across diet types for the contribution of minimally processed foods (defined earlier as Nova 1 and Nova 2 combined) and Nova 4 UPFs in daily food consumed (Fig. 2a, Fig. 3). However, the mean proportion of Nova 3 processed food consumption was the highest among regular red meat eaters (12.3%) and lower among those with plant-based dietary patterns especially among vegans (5.6%) (Fig. 2a).

The mean proportion of minimally processed food consumption was the lowest among regular red meat eaters (63.5%) and highest among vegans (71.7%). The mean UPF consumption was the lowest among pescatarians (20.4%) and highest among regular red meat eaters (24.2%). Compared with regular red meat eaters, vegans consumed a moderately larger amount of Nova 1 fruit, vegetables, legumes and nuts (Appendix Table S1). Several UPFs, including industrially processed breads, carbonated drinks, and ready meals, were commonly consumed by all diet types. However, the consumption of Nova 4 plant-based milk and meat alternatives were substantially higher among pescatarians, vegetarians and vegans (e.g., 4.9% vs 0.4% from plant-based milk, and 1.5% vs 0.03% from meat alternatives among vegans vs regular red meat eaters, respectively).

**Fig. 4:**
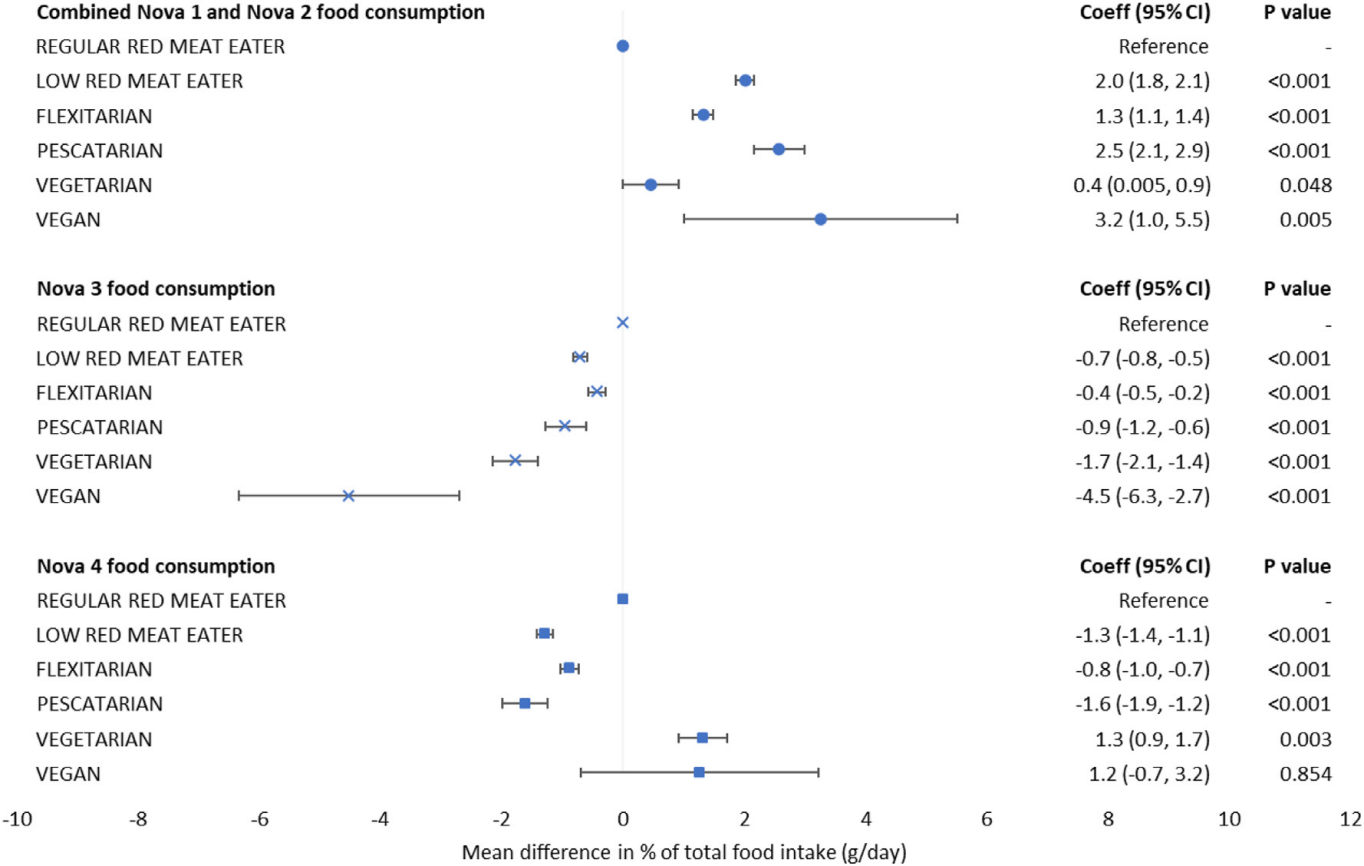
**Mean percentage points difference between diet types for the consumption of each Nova food group as measured by daily food intake.** Abbreviations: Coeff, coefficient; CI, confidence interval. Nova 1 includes unprocessed and minimally processed foods, Nova 2 includes processed culinary ingredients, Nova 3 represents processed foods, Nova 4 represents ultra-processed foods. All linear regression models were fully adjusted for age, sex, ethnicity, smoking status, physical activity, body mass index category, highest education attainment, average household income, Index of Multiple Derivation quintile, and total daily energy intake.

Results of fully adjusted regression models showed that compared with regular red meat eaters, all other diet types had a significantly larger mean consumption of minimally processed foods in their diet (Fig. 4). In particular, vegans consumed 3.2 percentage points (95% confidence interval [CI]: 1.0, 5.5) higher amount of minimally processed foods than regular red meat eaters. The consumption of UPFs showed a mixed pattern. Compared with regular red meat eaters, the mean UPF consumption were higher among vegetarians by 1.3 percentage points (95% CI: 0.9, 1.7) but lower among low red meat eaters, flexitarians, and pescatarians. There was no evidence of a difference in UPF consumption between vegans and regular red meat eaters.

### Dietary intakes based on the percentage of total energy (kcal/day) consumed

Based on the total daily energy consumed, the mean dietary contribution of minimally processed foods ranged from 36.1% in regular red meat eaters to 42.2% in vegans (Fig. 2b, Appendix Fig. S1 and Table S2). The mean dietary energy from UPFs were the lowest in pescatarians (46.4%) and highest in vegetarians (51.7%).

**Fig. 5:**
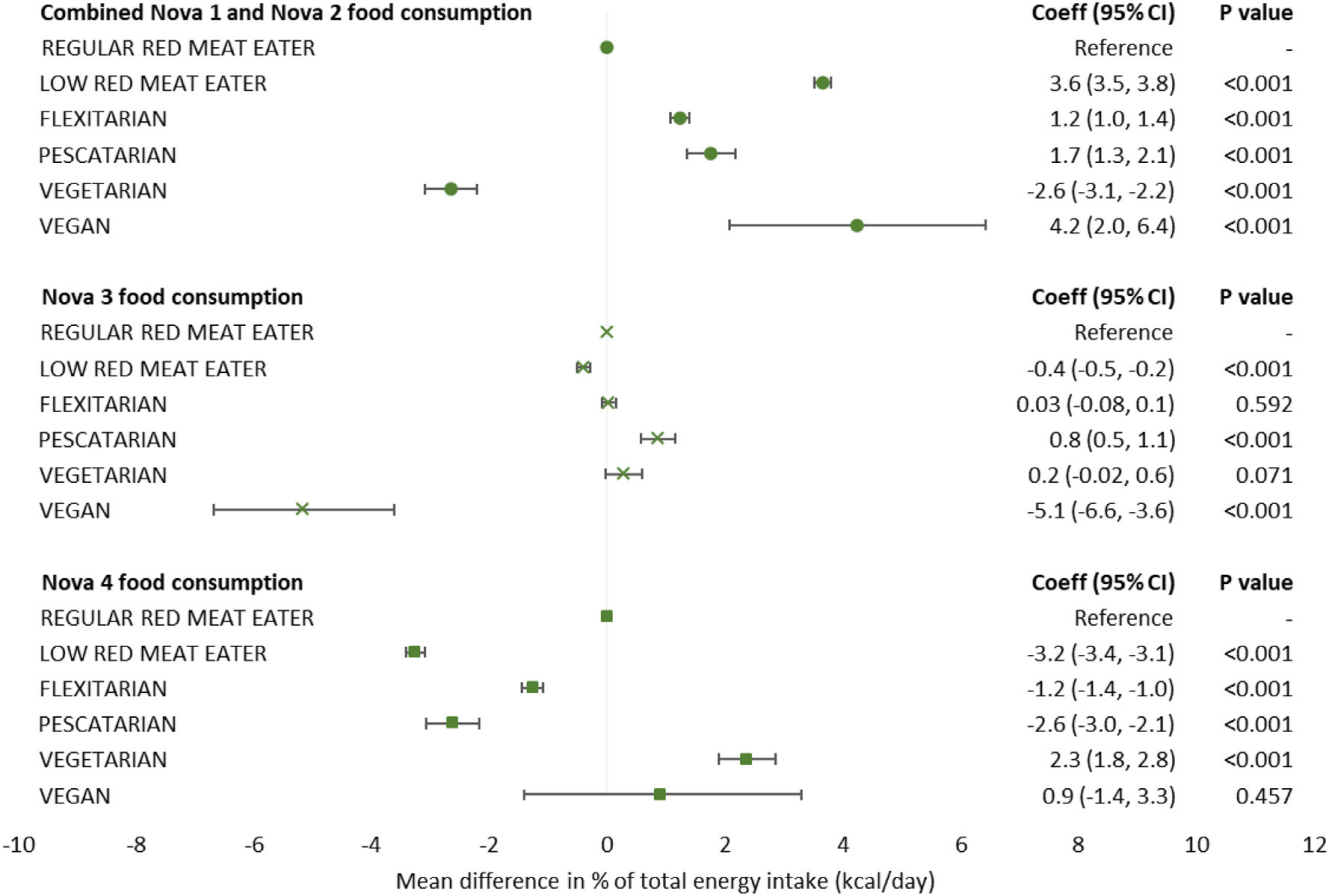
**Mean percentage points difference between diet types for the consumption of each Nova food group as measured by daily energy intake.** Abbreviations: Coeff, coefficient; CI, confidence interval. Nova 1 includes unprocessed and minimally processed foods, Nova 2 includes processed culinary ingredients, Nova 3 represents processed foods, Nova 4 represents ultra-processed foods. All linear regression models were fully adjusted for age, sex, ethnicity, smoking status, physical activity, body mass index category, highest education attainment, average household income, and Index of Multiple Derivation quintile.

Results from the fully adjusted regression models suggest that compared with regular red meat eaters, all other diet types had a significantly higher dietary energy sourced from minimally processed foods except for vegetarians (Fig. 5). Dietary energy sourced from UPFs were significantly lower among low red meat eaters, flexitarians, and pescatarians compared with regular red meat eaters. However, UPF consumption was significantly higher among vegetarians by 2.3 percentage points (95% CI: 1.8, 2.8) while no evidence of a difference was observed for vegans compared with regular red meat eaters.

### Consumption of Nova food groups measured as the amount in grams/calories consumed

The analyses of absolute food intake showed closely consistent results with that of the relative measures presented above except for the consumption of UPFs in vegans that was found significantly higher on average by 81.9 g/day (95% CI: 33.4, 130.4) compared with regular red meat eaters (Appendix Table S3). The absolute daily energy intake from minimally processed foods were significantly higher among low red meat eaters but lower among flexitarians, pescatarians, and vegetarians compared with regular red meat eaters. Moreover, the absolute daily energy sourced from UPFs were found significantly lower among all diet types compared with regular red meat eaters.

### Sensitivity analysis

The results of sensitivity analysis showed largely consistent findings particularly for the consumption of minimally processed foods and UPFs (Appendix Tables S4 and S5). There was an exception where analyses were restricted to participants with >1 dietary recalls (Appendix Table S4), where vegans were found consuming a similar proportion of minimally processed foods but a significantly higher proportion of UPFs than regular red meat eaters.

## Discussion

This large cross-sectional analysis of the UK Biobank study found no clear patterns for either UPF or minimally processed food consumption according to increasing levels of animal-sourced food avoidance. Consumption of UPFs was high in all diet types and represented more than 20% of daily food intake and more than 46% of daily energy intake among study participants. Vegans consumed on average 3.2 percentage points higher amount of minimally processed foods in their diets, but their UPF consumption was not found significantly different from those of regular red meat eaters. Furthermore, vegetarians consumed on average 1.3 percentage points higher amount of UPFs in their diet while low red meat eaters, flexitarians, and pescatarians consumed more than 0.8 percentage points lower amount of UPFs compared with regular red meat eaters.

Only two previous smaller studies assessed associations of diet types and UPF consumption.^24^^,^^25^ One study analysed consumption of a few selected UPFs in a German sample and found vegetarians were more likely to consume plant-based meat alternatives but less likely to consume fast food, sweet and salty snacks, and ultra-processed beverages compared with high meat eaters.^25^ The other study of a French sample analysed data collected using the same dietary assessment method as used in our study (24-h recall).^24^ The authors found higher UPF consumption in the diets of vegetarians and vegans than meat eaters (37.0%, 39.5%, and 33.0% of daily energy intake, respectively).^24^ By contrast, our findings did not show a consistent pattern of higher UPF consumption with greater levels of animal-sourced food avoidance. Our study found that compared with regular red meat eaters, low red meat eaters, flexitarians, and pescatarians ate a significantly lower amount of UPFs while vegetarians had a significantly higher UPF consumption. This was consistent across proportional consumption as measured by total food weight and total energy intake.

Although lower nutritional quality has been linked to ultra-processed dietary patterns,^8^ our study has shown higher fibre density and lower total energy, saturated fatty acids and sodium density among vegans. This may possibly be explained by the avoidance of meat and dairy, and higher consumptions of fruits, vegetables, legumes and nuts compared with other diet types. However, mean free sugar intake was universally high and above 13% of total energy intake for all diet types regardless of their level of animal-sourced food avoidance. This is more than double the maximum daily intake recommended in the UK and by the World Health Organization, and may be reflective of the high UPF consumption among all diet types.^26^^,^^27^

Dietary patterns represent a key opportunity for intervention on environmental sustainability given that the food system contributes to one third of global greenhouse gas emissions and consumes substantial natural resources.^7^ The planetary and human health co-benefits of a balanced diet based on fruit, vegetables, whole grains, legumes, and nuts are well-understood.^28^ However, plant-based diets vary in dietary quality when food ultra-processing is considered. UPFs are manufactured predominantly using cheap ingredients derived from a few high-yielding plants (e.g., corn, wheat, rice) or selected animal species fed on these crops in the case of animal-sourced UPFs.^8^^,^^29^ Any further increase in UPF consumption could worsen the already imbalanced food system and may accelerate biodiversity loss.^7^^,^^29^

Plant-based and low meat diets are often perceived as healthy and environmentally sustainable, and the increasing per capita consumption of meat and dairy alternatives in the UK and globally is reflective of the growing demand of plant-based alternatives.^5^ The UPF industry has responded to these demands as evident from the increased production and marketing of new ranges of plant-based UPF products every year.^17^ However, novel evidence from literature has suggested that the degree and purpose of food processing might be important when considering the healthfulness of plant-based foods.^6^^,^^16^ In addition to evidence from well-conducted prospective cohort studies that has shown links between UPF consumption and obesity, type 2 diabetes, and various negative health outcomes, emerging research has demonstrated that plant-sourced UPFs were detrimental for cardiovascular health.^11^, ^12^, ^13^, ^14^, ^15^, ^16^ In this study, we found that UPF consumption were universally high in the diets of UK Biobank participants, and many UPFs, including mass-produced bakery goods, carbonated drinks, and ready-to-eat foods, were commonly consumed by all diet types. Furthermore, our findings highlight that pescatarians, vegetarians, and vegans were more likely to include plant-based milk and meat alternatives in their diet. This is concerning as UPFs produced purely from plant-derived substances are increasingly promoted by the UPF industry as healthy and sustainable alternatives to mobilise consumers’ transition away from meat-based diets. It is, therefore, important that urgently needed policies that address food system sustainability also promote rebalancing diets towards minimally processed foods away from UPFs.

Our study has many strengths. We present a comprehensive analysis of dietary consumption based on both weight and energy content of all foods and drinks consumed. The large sample size enables comparison between six dietary patterns, including by the type and level of animal-sourced food avoidance. The use of both FFQ and 24-h recall data facilitates more accurate categorization of diet types. The validated 24-h recalls allowed for more detailed dietary consumption data being captured than many FFQs.

There are important limitations to acknowledge. First, the study population was not nationally representative and may over-represent populations with white ethnicity, and the mean UPF consumption were lower than UK average.^9^ Second, the relatively small number of vegans may have contributed to the large uncertainties in the regression coefficients estimated for this diet type. Third, misclassification bias may be present for a few food items due to limited information provided on food processing. We have taken the approach assigning them to the most probable food group based on published findings of common foods and drinks consumed in the UK.^9^ Fourth, the study is limited by 39.6% of the sample with only one 24-h recall, but our sensitivity analysis restricting to samples with >1 dietary recalls showed largely similar findings. Fifth, the 24-h recall data were completed by the UK Biobank participants during 2009–2012. Thus, the dietary patterns compared in this study may not fully reflect the increasing availability and sales of plant-sourced UPF products on the market.^5^ Sixth, the self-completed FFQ and 24-h recall data could be subject to potential mis-reporting, social desirability and recall bias. Finally, owing to the observational nature of the study, any residual confounding may bias the findings although a wide range of socio-demographic and lifestyle factors were adjusted for.

This large UK-based study found similarly high UPF consumption among diet types regardless of their levels of animal-sourced food avoidance. Policies that encourage the urgently needed transition to more sustainable and plant-based dietary patterns should promote the consumption of minimally processed foods, and rebalancing diets away from UPFs.

## Contributors

KC, JCP, and EPV conceptualised the study. KC compiled the data and performed statistical analyses. KC and EPV had access and verified the underlying data used in this study. All authors contributed to the finalisation of statistical models and interpretation of findings. KC and EPV wrote the first draft of the manuscript, and JCP, FR, RBL, IH, MJG, and CM critically reviewed and edited the manuscript. All authors had full access to all the data in the study, approved the final manuscript, and accept responsibility for the decision to submit for publication.

## Data sharing statement

UK Biobank data are available through application to the database https://www.ukbiobank.ac.uk/.

## Declaration of interests

All authors declare no conflict of interest.
