# Supplementary material for: Plant-based dietary patterns and ultra-processed food consumption: a cross-sectional analysis of the UK Biobank
Source: eClinicalMedicine. 2024 Nov 14;78:102931. doi: 10.1016/j.eclinm.2024.102931 (PMC11605131; doi:10.1016/j.eclinm.2024.102931)
Supplement: Appendix Fig. S1 and Tables S1–S5 [file mmc1.pdf]

Supplementary material

Plant-based dietary patterns and ultra-processed food consumption: a cross-sectional analysis of the UK Biobank

Kiara Chang, Jennie C Parnham, Fernanda Rauber, Renata B Levy, Inge Huybrechts, Marc J Gunter, Christopher Millett, Eszter P Vamos

Table of Contents

Appendix Figure 1: Mean proportion of daily energy intake from subsidiary food groups of: a) Nova 1; b) Nova 2 and Nova 3; and c) Nova 4 food group 2

Appendix Table 1: Mean dietary proportion of daily food intake by subsidiary food groups of Nova 3

Appendix Table 2: Mean dietary proportion of daily energy intake by subsidiary food groups of Nova 4

Appendix Table 3: Multivariable linear regression for the secondary outcome measures on the consumption of each Nova food group 5

Appendix Table 4: Sensitivity analysis for the mean percentage points difference between diet types as measured by daily food intake 6

Appendix Table 5: Sensitivity analysis for the mean proportion of daily energy intake 7

Appendix Figure 1: Mean proportion of daily energy intake from subsidiary food groups of: a) Nova 1; b) Nova 2 and Nova 3; and c) Nova 4 food group

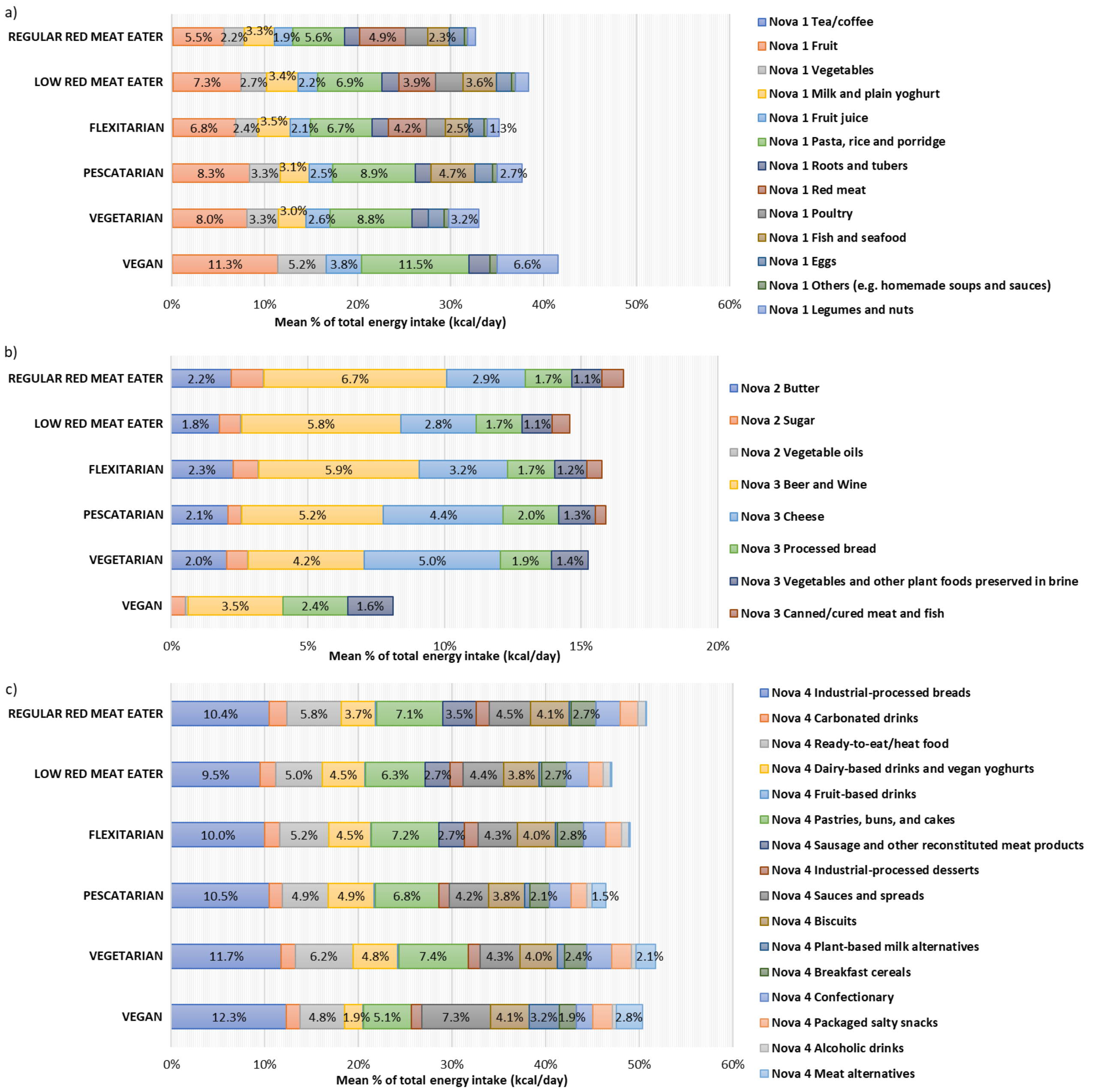

Nova 1 includes unprocessed and minimally processed foods, Nova 2 includes processed culinary ingredients, Nova 3 represents processed foods, Nova 4 represents ultra-processed foods.

Appendix Table 1: Mean dietary proportion of daily food intake by subsidiary food groups of Nova

	Regular red meat eater<br>Mean (SE)<br>% daily food intake	Low red meat eater<br>Mean (SE)<br>% daily food intake	Flexitarian<br>Mean (SE)<br>% daily food intake	Pescatarian<br>Mean (SE)<br>% daily food intake	Vegetarian<br>Mean (SE)<br>% daily food intake	Vegan<br>Mean (SE)<br>% daily food intake
**Nova 1 Unprocessed/minimally processed food**	62.9 (0.06)	68.1 (0.05)‡	67.4 (0.07)‡	70.4 (0.2)‡	67.9 (0.2)‡	71.5 (1.1)‡
Tea/coffee	28.1 (0.06)	29.0 (0.06)‡	30.1 (0.07)‡	31.2 (0.2)‡	30.0 (0.2)‡	29.6 (1.3)
Fruit	7.3 (0.02)	9.1 (0.02)‡	8.6 (0.03)‡	9.6 (0.09)‡	9.5 (0.1)‡	11.4 (0.6)‡
Vegetables	6.1 (0.01)	7.4 (0.02)‡	6.6 (0.02)‡	8.5 (0.09)‡	8.8 (0.1)‡	12.7 (0.6)‡
Milk and plain yoghurt	6.0 (0.02)	6.0 (0.02)	6.3 (0.02)‡	5.3 (0.08)‡	5.1 (0.09)‡	-
Fruit juice	4.1 (0.02)	4.2 (0.02)‡	4.3 (0.02)‡	4.8 (0.08)‡	5.0 (0.10)‡	6.7 (0.6)‡
Pasta, rice and porridge	3.1 (0.01)	3.7 (0.01)‡	3.5 (0.01)‡	4.4 (0.06)‡	4.5 (0.07)‡	5.7 (0.4)‡
Roots and tubers	1.7 (0.01)	1.8 (0.01)‡	1.8 (0.01)‡	1.7 (0.04)	1.7 (0.04)	2.0 (0.2)
Red meat	2.0 (0.009)	1.5 (0.009)‡	1.6 (0.01)‡	-	-	-
Poultry	1.2 (0.008)	1.5 (0.009)‡	1.0 (0.009)‡	-	-	-
Fish and seafood	0.9 (0.007)	1.4 (0.009)‡	1.0 (0.01)‡	1.8 (0.04)‡	-	-
Eggs	0.8 (0.006)	0.8 (0.007)‡	0.8 (0.008)‡	0.9 (0.02)†	0.8 (0.02)‡	-
Others (e.g. homemade soups and sauces)	0.6 (0.008)	0.8 (0.009)‡	0.8 (0.01)‡	1.0 (0.03)‡	0.9 (0.03)‡	0.9 (0.1)
Legumes and nuts	0.2 (0.003)	0.4 (0.004)‡	0.3 (0.004)‡	0.8 (0.01)‡	1.1 (0.02)‡	2.1 (0.1)‡
**Nova 2 Processed culinary ingredient**	0.5 (0.003)	0.3 (0.002)‡	0.4 (0.003)‡	0.3 (0.008)‡	0.3 (0.01)‡	0.1 (0.02)‡
Butter	0.2 (0.002)	0.1 (0.002)‡	0.2 (0.002)‡	0.2 (0.006)‡	0.2 (0.007)‡	-
Sugar	0.2 (0.002)	0.1 (0.002)‡	0.1 (0.003)‡	0.09 (0.004)‡	0.1 (0.007)‡	0.1 (0.02)‡
Vegetable oils	0.001 (0.00008)	0.002 (0.00009)‡	0.002 (0.00001)‡	0.002 (0.0004)‡	0.002 (0.0005)‡	0.006 (0.004)†
**Nova 3 Processed food**	12.2 (0.05)	9.6 (0.04)‡	10.0 (0.05)‡	8.8 (0.1)‡	7.8 (0.1)‡	5.5 (0.7)‡
Beer and Wine	10.3 (0.05)	7.8 (0.04)‡	8.1 (0.05)‡	6.6 (0.1)‡	5.8 (0.1)‡	4.5 (0.7)‡
Cheese	0.6 (0.003)	0.6 (0.003)‡	0.7 (0.004)‡	0.9 (0.01)‡	1.0 (0.01)‡	-
Processed bread	0.5 (0.006)	0.4 (0.005)	0.5 (0.007)	0.5 (0.01)‡	0.5 (0.02)‡	0.5 (0.1)
Vegetables and other plant foods preserved in brine	0.3 (0.002)	0.3 (0.003)‡	0.3 (0.01)	0.4 (0.01)‡	0.4 (0.05)‡	0.4 (0.003)‡
Canned/cured meat and fish	0.3 (0.003)	0.3 (0.003)‡	0.2 (0.004)‡	0.2 (0.01)‡	-	-
**Nova 4 Ultra-processed food**	24.2 (0.05)	21.8 (0.04)‡	22.0 (0.06)‡	20.4 (0.1)‡	23.8 (0.2)†	22.7 (1.0)
Industrial-processed breads	3.5 (0.01)	3.0 (0.009)‡	3.3 (0.01)‡	3.2 (0.03)‡	3.7 (0.04)	3.8 (0.2)
Carbonated drinks	3.5 (0.02)	2.8 (0.02)‡	2.8 (0.03)‡	2.3 (0.07)‡	2.6 (0.09)‡	2.4 (0.5)‡
Ready-to-eat/heat food	3.2 (0.01)	2.7 (0.01)‡	2.8 (0.02)‡	2.5 (0.05)‡	3.2 (0.07)‡	2.4 (0.2)
Dairy-based drinks	2.3 (0.01)	2.9 (0.01)‡	2.7 (0.02)‡	2.8 (0.06)‡	3.0 (0.07)‡	1.0 (0.1)†
Fruit-based drinks	2.9 (0.02)	2.7 (0.02)‡	2.1 (0.02)†	1.9 (0.08)‡	2.3 (0.1)	1.3 (0.3)‡
Pastries, buns, and cakes	1.9 (0.009)	1.6 (0.009)‡	1.8 (0.01)‡	1.6 (0.03)‡	1.9 (0.04)‡	1.3 (0.1)‡
Sausage and other reconstituted meat products	1.3 (0.008)	0.9 (0.007)‡	1.0 (0.009)‡	-	-	-
Industrial-processed desserts	1.0 (0.006)	0.9 (0.006)‡	1.0 (0.009)†	0.7 (0.02)‡	0.8 (0.02)‡	0.9 (0.1)
Sauces and spreads	1.1 (0.006)	1.1 (0.007)‡	1.0 (0.008)‡	0.9 (0.02)‡	1.0 (0.02)‡	1.0 (0.08)
Biscuits	0.7 (0.004)	0.6 (0.004)‡	0.7 (0.006)‡	0.6 (0.01)‡	0.7 (0.01)‡	0.7 (0.1)‡
Plant-based milk alternatives	0.3 (0.007)	0.5 (0.008)‡	0.4 (0.01)‡	1.0 (0.04)‡	1.3 (0.06)‡	4.8 (0.4)‡
Breakfast cereals	0.6 (0.003)	0.5 (0.004)‡	0.6 (0.004)	0.4 (0.01)‡	0.5 (0.01)‡	0.3 (0.04)‡
Confectionary	0.4 (0.004)	0.4 (0.003)‡	0.4 (0.005)‡	0.3 (0.01)‡	0.4 (0.01)	0.2 (0.04)†
Packaged salty snacks	0.3 (0.002)	0.2 (0.002)‡	0.2 (0.003)‡	0.2 (0.008)‡	0.3 (0.01)†	0.3 (0.04)
Alcoholic drinks	0.3 (0.004)	0.2 (0.004)‡	0.2 (0.004)‡	0.1 (0.01)‡	0.1 (0.01)‡	0.1 (0.03)‡
Sweeteners	0.1 (0.002)	0.1 (0.002)‡	0.1 (0.002)‡	0.06 (0.005)‡	0.07 (0.006)‡	0.03 (0.01)‡
Meat alternatives	0.03 (0.001)	0.06 (0.002)‡	0.08 (0.002)‡	0.8 (0.02)‡	1.2 (0.02)‡	1.5 (0.1)‡

Abbreviations: SE, standard error.
†p<0.05; ‡p<0.01 from ranksum test comparing distribution of consumption against regular red meat eaters.

Appendix Table 2: Mean dietary proportion of daily energy intake by subsidiary food groups of Nova

	Regular red meat eater	Low red meat eater	Flexitarian	Pescatarian	Vegetarian	Vegan
	Mean (SE) % daily energy intake	Mean (SE) % daily energy intake	Mean (SE) % daily energy intake	Mean (SE) % daily energy intake	Mean (SE) % daily energy intake	Mean (SE) % daily energy intake
**Nova 1 Unprocessed/minimally processed food**	32.7 (0.05)	38.3 (0.05)‡	35.2 (0.06)‡	37.7 (0.2)‡	33.0 (0.2)	41.5 (1.3)‡
Tea/coffee	0.1 (0.0006)	0.1 (0.0006)‡	0.1 (0.0008)‡	0.08 (0.002)‡	0.09 (0.003)‡	0.07 (0.07)‡
Fruit	5.5 (0.01)	7.3 (0.02)‡	6.7 (0.02)‡	8.3 (0.08)‡	8.0 (0.1)‡	11.3 (0.6)‡
Vegetables	2.1 (0.007)	2.7 (0.009)‡	2.4 (0.01)‡	3.2 (0.04)‡	3.3 (0.04)‡	5.2 (0.3)‡
Milk and plain yoghurt	3.2 (0.01)	3.3 (0.01)‡	3.5 (0.01)‡	3.0 (0.05)‡	2.9 (0.05)‡	-
Fruit juice	1.9 (0.01)	2.1 (0.01)‡	2.1 (0.01)‡	2.5 (0.04)‡	2.5 (0.05)‡	3.8 (0.4)‡
Pasta, rice and porridge	5.5 (0.02)	6.8 (0.03)‡	6.6 (0.1)‡	8.8 (0.1)‡	8.8 (0.7)‡	11.5 (0.01)‡
Roots and tubers	1.6 (0.009)	1.7 (0.01)‡	1.7 (0.01)‡	1.7 (0.03)†	1.7 (0.04)†	2.2 (0.2)
Red meat	4.9 (0.02)	3.9 (0.02)‡	4.1 (0.02)‡	-	-	-
Poultry	2.3 (0.01)	2.9 (0.01)‡	2.0 (0.01)‡	-	-	-
Fish and seafood	2.3 (0.01)	3.5 (0.02)‡	2.5 (0.02)‡	4.6 (0.1)‡	-	-
Eggs	1.6 (0.01)	1.6 (0.01)‡	1.5 (0.01)‡	1.9 (0.05)‡	1.6 (0.05)‡	-
Others (e.g. homemade soups and sauces)	0.3 (0.005)	0.3 (0.005)‡	0.3 (0.007)‡	0.5 (0.02)‡	0.4 (0.02)‡	0.7 (0.1)†
Legumes and nuts	0.8 (0.008)	1.4 (0.01)‡	1.2 (0.01)‡	2.7 (0.06)‡	3.2 (0.07)‡	6.5 (0.5)‡
**Nova 2 Processed culinary ingredient**	3.3 (0.01)	2.5 (0.01)‡	3.1 (0.02)‡	2.5 (0.05)‡	2.8 (0.06)‡	0.6 (0.1)‡
Butter	2.1 (0.01)	1.7 (0.01)‡	2.2 (0.01)‡	2.0 (0.05)‡	2.0 (0.05)‡	-
Sugar	1.1 (0.01)	0.7 (0.008)‡	0.9 (0.01)‡	0.4 (0.02)‡	0.7 (0.03)‡	0.5 (0.1)‡
Vegetable oils	0.01 (0.001)	0.02 (0.001)‡	0.02 (0.001)‡	0.03 (0.005)‡	0.02 (0.005)‡	0.09 (0.05)†
**Nova 3 Processed food**	13.1 (0.03)	12.0 (0.03)‡	12.5 (0.04)‡	13.3 (0.1)‡	12.4 (0.1)‡	7.4 (0.7)‡
Beer and Wine	6.6 (0.03)	5.8 (0.02)‡	5.8 (0.03)‡	5.1 (0.09)‡	4.2 (0.1)‡	3.4 (0.5)‡
Cheese	2.8 (0.01)	2.7 (0.01)‡	3.2 (0.1)‡	4.3 (0.07)‡	4.9 (0.07)‡	-
Processed bread	1.7 (0.01)	1.6 (0.01)	1.7 (0.02)‡	2.0 (0.06)‡	1.8 (0.06)‡	2.3 (0.4)
Vegetables and other plant foods preserved in brine	1.1 (0.008)	1.1 (0.008)‡	1.1 (0.01)‡	1.3 (0.03)‡	1.3 (0.03)‡	1.6 (0.2)‡
Canned/cured meat and fish	0.7 (0.006)	0.6 (0.006)‡	0.5 (0.007)‡	0.3 (0.01)‡	-	-
**Nova 4 Ultra-processed food**	50.7 (0.05)	47.0 (0.06)‡	48.9 (0.07)‡	46.3 (0.2)‡	51.7 (0.2)‡	50.3 (1.3)
Industrial-processed breads	10.4 (0.02)	9.5 (0.02)‡	9.9 (0.03)‡	10.4 (0.1)	11.6 (0.1)‡	12.2 (0.7)†
Carbonated drinks	1.9 (0.01)	1.6 (0.01)‡	1.6 (0.01)‡	1.3 (0.04)‡	1.5 (0.05)‡	1.4 (0.3)‡
Ready-to-eat/heat food	5.7 (0.03)	4.9 (0.02)‡	5.2 (0.03)‡	4.8 (0.1)‡	6.1 (0.1)‡	4.7 (0.5)
Dairy-based drinks	3.6 (0.03)	4.5 (0.03)‡	4.4 (0.03)‡	4.9 (0.1)‡	4.7 (0.1)‡	1.9 (0.3)‡
Fruit-based drinks	0.1 (0.001)	0.1 (0.001)‡	0.09 (0.001)‡	0.09 (0.005)‡	0.1 (0.005)‡	0.07 (0.02)†
Pastries, buns, and cakes	7.0 (0.03)	6.3 (0.03)‡	7.2 (0.04)	6.8 (0.1)‡	7.4 (0.1)	5.1 (0.5)‡
Sausage and other reconstituted meat products	3.5 (0.02)	2.6 (0.02)‡	2.6 (0.02)‡	-	-	-
Industrial-processed desserts	1.4 (0.009)	1.3 (0.009)‡	1.4 (0.01)	1.1 (0.03)‡	1.2 (0.03)‡	1.1 (0.1)†
Sauces and spreads	4.4 (0.01)	4.3 (0.01)‡	4.2 (0.01)‡	4.1 (0.06)‡	4.3 (0.06)‡	7.2 (0.4)‡
Biscuits	4.1 (0.02)	3.7 (0.02)‡	4.0 (0.02)‡	3.8 (0.08)‡	3.9 (0.09)‡	4.1 (0.6)‡
Plant-based alternatives	0.1 (0.003)	0.2 (0.004)‡	0.2 (0.005)‡	0.5 (0.02)‡	0.7 (0.03)‡	3.2 (0.2)‡
Breakfast cereals	2.6 (0.01)	2.7 (0.01)†	2.7 (0.01)	2.1 (0.04)‡	2.4 (0.05)‡	1.8 (0.2)‡
Confectionary	2.5 (0.01)	2.3 (0.01)‡	2.4 (0.02)‡	2.2 (0.06)‡	2.6 (0.08)	1.6 (0.2)†
Packaged salty snacks	1.8 (0.01)	1.5 (0.01)‡	1.6 (0.01)‡	1.7 (0.04)‡	2.0 (0.05)‡	2.1 (0.3)
Alcoholic drinks	0.8 (0.01)	0.7 (0.01)‡	0.7 (0.01)‡	0.5 (0.03)‡	0.4 (0.03)‡	0.3 (0.1)‡
Sweeteners	0 (0)	0 (0)	0 (0)	0 (0)	0 (0)	0 (0)
Meat alternatives	0.06 (0.002)	0.1 (0.003)‡	0.1 (0.005)‡	1.4 (0.03)‡	2.1 (0.04)‡	2.8 (0.2)‡

Abbreviations: SE, standard error.
†$p<0.05$; ‡$p<0.01$ from ranksum test comparing distribution of consumption against regular red meat eaters.

Appendix Table 3: Multivariable linear regression for the secondary outcome measures on the consumption of each Nova food group

	**Mean food intake** g/day	P value	**Mean energy intake** kcal/day	P value
**Nova 1 and Nova 2 food consumption combined**				
Regular red meat eater	[Reference]		[Reference]	
Low red meat eater	85.8 (79.2, 92.3)	<0.001	26.6 (23.4, 29.7)	<0.001
Flexitarian	20.8 (13.4, 28.1)	<0.001	-23.2 (-26.7, -19.7)	<0.001
Pescatarian	140.0 (122.0, 158.0)	<0.001	-11.8 (-20.4, -3.2)	0.007
Vegetarian	53.6 (34.0, 73.1)	<0.001	-108.5 (-117.9, -99.1)	<0.001
Vegan	234.7 (138.4, 330.9)	<0.001	-40.7 (-86.8, 5.4)	0.084
**Nova 3 food consumption**				
Regular red meat eater	[Reference]		[Reference]	
Low red meat eater	-22.2 (-26.4, -18.0)	<0.001	-25.0 (-27.3, -22.6)	<0.001
Flexitarian	-19.2 (-24.0, -14.5)	<0.001	-17.6 (-20.2, -14.9)	<0.001
Pescatarian	-23.2 (-34.8, -11.5)	<0.001	0.8 (-5.6, 7.2)	0.809
Vegetarian	-42.5 (-55.2, -29.9)	<0.001	-9.7 (-16.8, -2.6)	0.007
Vegan	-108.4 (-170.5, -46.2)	0.001	-129.8 (-164.4, -95.1)	<0.001
**Nova 4 food consumption**				
Regular red meat eater	[Reference]		[Reference]	
Low red meat eater	-21.8 (-25.1, -18.5)	<0.001	-129.5 (-134.7, -124.2)	<0.001
Flexitarian	-30.7 (-34.4, -27.0)	<0.001	-94.2 (-100.1, -88.2)	<0.001
Pescatarian	-21.8 (-30.8, -12.7)	<0.001	-109.6 (-124.2, -95.1)	<0.001
Vegetarian	40.9 (31.1, 50.8)	<0.001	-18.8 (-34.7, -3.0)	0.019
Vegan	81.9 (33.4, 130.4)	0.001	-113.8 (-101.5, -36.0)	0.004

Linear regression models were fully adjusted for age, sex, ethnicity, smoking status, physical activity, body mass index category, highest education attainment, average household income, Index of Multiple Derivation quintile, and total daily energy intake. Nova 1 includes unprocessed and minimally processed foods, Nova 2 includes processed culinary ingredients, Nova 3 represents processed foods, Nova 4 represents ultra-processed foods.

Appendix Table 4: Sensitivity analysis for the mean percentage points difference between diet types as measured by daily food intake

	Model S1		Model S2		Model S3	
	% daily food intake	P value	% daily food intake	P value	%g daily food intake	P value
**Nova 1 and Nova 2 food consumption combined**						
Regular red meat eater	[Reference]		[Reference]		[Reference]	
Low red meat eater	2.1 (2.0, 2.3)	<0.001	2.0 (1.8, 2.1)	<0.001	1.8 (1.6, 1.9)	<0.001
Flexitarian	1.6 (1.4, 1.8)	<0.001	1.3 (1.1, 1.4)	<0.001	1.3 (1.1, 1.5)	<0.001
Pescatarian	3.2 (2.8, 3.6)	<0.001	2.5 (2.1, 2.9)	<0.001	2.4 (1.9, 2.9)	<0.001
Vegetarian	1.1 (0.6, 1.5)	<0.001	0.4 (0.01, 0.9)	0.045	0.7 (0.1, 1.2)	<0.001
Vegan	4.2 (1.9, 6.5)	<0.001	3.2 (0.9, 5.5)	0.005	2.2 (-0.2, 4.7)	0.078
**Nova 3 food consumption**						
Regular red meat eater	[Reference]		[Reference]		[Reference]	
Low red meat eater	-0.7 (-0.8, -0.5)	<0.001	-0.7 (-0.8, -0.5)	<0.001	-0.7 (-0.8, -0.5)	<0.001
Flexitarian	-0.4 (-0.5, -0.2)	<0.001	-0.4 (-0.5, -0.2)	<0.001	-0.3 (-0.5, -0.2)	<0.001
Pescatarian	-0.9 (-1.3, -0.6)	<0.001	-0.9 (-1.2, -0.6)	<0.001	-0.8 (-1.2, -0.4)	<0.001
Vegetarian	-1.8 (-2.1, -1.4)	<0.001	-1.7 (-2.1, -1.4)	<0.001	-1.9 (-2.3, -1.4)	<0.001
Vegan	-4.5 (-6.3, -2.7)	<0.001	-4.5 (-6.3, -2.7)	<0.001	-4.8 (-6.8, -2.8)	<0.001
**Nova 4 food consumption**						
Regular red meat eater	[Reference]		[Reference]		[Reference]	
Low red meat eater	-1.4 (-1.5, -1.3)	<0.001	-1.2 (-1.4, -1.1)	<0.001	-1.1 (-1.2, -0.9)	<0.001
Flexitarian	-1.2 (-1.3, -1.0)	<0.001	-0.8 (-1.0, -0.7)	<0.001	-1.0 (-1.1, -0.8)	<0.001
Pescatarian	-2.2 (-2.6, -1.9)	<0.001	-1.6 (-1.9, -1.2)	<0.001	-1.5 (-1.9, -1.1)	<0.001
Vegetarian	0.7 (0.3, 1.1)	0.001	1.3 (0.9, 1.7)	<0.001	1.2 (0.7, 1.6)	<0.001
Vegan	0.3 (-1.6, 2.2)	0.767	1.2 (-0.6, 3.2)	0.201	2.6 (0.4, 4.7)	0.016

Linear regression models were fully adjusted for age, sex, ethnicity, smoking status, physical activity, body mass index category, highest education attainment, average household income, Index of Multiple Derivation quintile, and total daily energy intake.
Nova 1 includes unprocessed and minimally processed foods, Nova 2 includes processed culinary ingredients, Nova 3 represents processed foods, Nova 4 represents ultra-processed foods.
Model S1 excluded body mass index from study covariates.
Model S2 additionally adjusted for self-reported diabetes, high blood pressure, cardiovascular disease, and depression.
Model S3 excluded participants with <2 dietary recalls (N=120,594).

Appendix Table 5: Sensitivity analysis for the mean proportion of daily energy intake

	Model S1 % daily energy intake	P value	Model S2 % daily energy intake	P value	Model S3 % daily energy intake	P value
**Nova 1 and Nova 2 food consumption combined**						
Regular red meat eater	[Reference]		[Reference]		[Reference]	
Low red meat eater	3.7 (3.5, 3.8)	<0.001	3.6 (3.5, 3.8)	<0.001	3.4 (3.2, 3.5)	<0.001
Flexitarian	1.3 (1.1, 1.4)	<0.001	1.2 (1.0, 1.4)	<0.001	1.2 (1.0, 1.4)	<0.001
Pescatarian	1.9 (1.5, 2.3)	<0.001	1.7 (1.3, 2.1)	<0.001	1.5 (1.0, 1.9)	<0.001
Vegetarian	-2.4 (-2.9, -2.0)	<0.001	-2.6 (-3.0, -2.1)	<0.001	-2.5 (-3.0, -2.1)	<0.001
Vegan	4.5 (2.3, 6.7)	<0.001	4.2 (2.1, 6.4)	<0.001	4.0 (1.6, 6.3)	0.001
**Nova 3 food consumption**						
Regular red meat eater	[Reference]		[Reference]		[Reference]	
Low red meat eater	-0.3 (-0.4, -0.2)	<0.001	-0.4 (-0.5, -0.3)	<0.001	-0.3 (-0.4, -0.2)	<0.001
Flexitarian	0.06 (-0.05, 0.1)	0.295	0.03 (-0.09, 0.1)	0.604	0.1 (0.003, 0.2)	0.045
Pescatarian	0.9 (0.6, 1.1)	<0.001	0.8 (0.5, 1.1)	<0.001	0.9 (0.6, 1.2)	<0.001
Vegetarian	0.3 (0.01, 0.6)	0.041	0.3 (-0.01, 0.6)	0.062	0.2 (-0.1, 0.5)	0.176
Vegan	-5.0 (-6.6, -3.5)	<0.001	-5.1 (-6.6, -3.6)	<0.001	-5.7 (-7.3, -4.0)	<0.001
**Nova 4 food consumption**						
Regular red meat eater	[Reference]		[Reference]		[Reference]	
Low red meat eater	-3.3 (-3.4, -3.1)	<0.001	-3.2 (-3.4, -3.0)	<0.001	-3.0 (-3.2, -2.8)	<0.001
Flexitarian	-1.3 (-1.5, -1.2)	<0.001	-1.2 (-1.4, -1.0)	<0.001	-1.3 (-1.5, -1.1)	<0.001
Pescatarian	-2.8 (-3.3, -2.4)	<0.001	-2.6 (-3.0, -2.2)	<0.001	-2.4 (-2.9, -1.9)	<0.001
Vegetarian	2.1 (1.6, 2.6)	<0.001	2.3 (1.8, 2.8)	<0.001	2.3 (1.8, 2.8)	<0.001
Vegan	0.5 (-1.8, 2.9)	0.643	0.8 (-1.5, 3.2)	0.482	1.7 (-0.8, 4.2)	0.195

Linear regression models were fully adjusted for age, sex, ethnicity, smoking status, physical activity, body mass index category, highest education attainment, average household income, and Index of Multiple Derivation quintile. Nova 1 includes unprocessed and minimally processed foods, Nova 2 includes processed culinary ingredients, Nova 3 represents processed foods, Nova 4 represents ultra-processed foods.
Model S1 excluded body mass index from study covariates.
Model S2 additionally adjusted for self-reported diabetes, high blood pressure, cardiovascular disease, and depression.
Model S3 excluded participants with <2 dietary recalls (N=120,594).
